# Why Are Some Population Interventions for Diet and Obesity More Equitable and Effective Than Others? The Role of Individual Agency

**DOI:** 10.1371/journal.pmed.1001990

**Published:** 2016-04-05

**Authors:** Jean Adams, Oliver Mytton, Martin White, Pablo Monsivais

**Affiliations:** Centre for Diet and Activity Research, MRC Epidemiology Unit, University of Cambridge, Cambridge, United Kingdom

## Abstract

Jean Adams and colleagues argue that population interventions that require individuals to use a low level of agency to benefit are likely to be most effective and most equitable.

**Summary Points**

- Public health interventions can be described according to where they lie on two continuums: the population and high-risk approaches anchor one continuum, while the other continuum captures the personal resources (or “agency”) individuals have to use to benefit from interventions.
- Population interventions that require individuals to use a high level of agency to benefit tend to be favoured by governments around the world.
- Population interventions that require individuals to use a low level of agency to benefit are likely to be most effective and most equitable. More effort is required to develop, evaluate, and implement population interventions that require low levels of agency for individuals to benefit.

We now find ourselves in a world in which one in three adults is overweight or obese [1]. Unhealthy diets are an important determinant of overweight and obesity and also contribute directly to a variety of other causes of disability and death. In fact, together with physical inactivity, dietary risk factors are responsible for 10% of disability-adjusted life years lost globally [2]. Like many risk factors, unhealthy diets are not evenly distributed in the population. In most developed countries, and increasingly in developing countries too, obesity and unhealthy diets are more common in more socioeconomically disadvantaged groups [3,4]. Socioeconomic inequalities in health and disease are at least partly due to these inequalities in diet and obesity.

Many governments have responded to the challenge of unhealthy diets and obesity with strategies that focus on advice, guidance, and encouragement to adopt healthier lifestyles. In England, the government’s current “flagship” obesity prevention programme is Change4Life. This social marketing campaign uses mass media and other avenues to inform and educate the public about the harms of excess weight and the benefits of being more active and eating more healthfully. Simple strategies for changing behaviour are also offered. Similar programmes operate elsewhere, including MangerBouger in France and Let’s Move in the United States.

The obvious assumption of these programmes is that advice, guidance, and encouragement will change the population’s diet and activity behaviours. We explore why this is unlikely to be the case and why such strategies are unlikely to reduce inequalities in diet and obesity. We propose a framework that captures a wider range of intervention strategies and discuss how these may be used to improve diet, reduce obesity, and tackle inequalities.

## The Population and High-Risk Approaches to Prevention

Mass-media social marketing initiatives draw on Geoffrey Rose’s population approach to prevention [5]. This approach involves interventions that are delivered across the whole population, or whole population groups, without first identifying those at increased risk of disease. The aim of the population approach is to reduce risk factor levels by a small amount in everyone. In contrast, the high-risk approach identifies and targets those individuals at high risk of disease, aiming to reduce risk factors by a large amount in only these people. Rose argued that a greater overall benefit would be achieved by modest reductions in risk in many people than by large reductions in just a few people.

Unfortunately, not all population approaches are the same [6]. Population approaches that rely on individuals engaging with information are unlikely to achieve the full potential of Rose’s insights.

## Population Interventions Differ in the Degree of “Agency” Individuals Must Use to Benefit

Population interventions like Change4Life that focus on providing advice, guidance, and encouragement rely heavily on individuals being able and motivated to engage with this advice, guidance, and encouragement. These types of interventions have been described as highly “agentic” [7,8]: recipients must use their personal resources, or “agency,” to benefit. The effectiveness and equity of these interventions has been questioned [7,8].

In contrast, population interventions that require recipients to use little or no agency to benefit may be more effective and equitable. When food manufacturers reduce the salt content of bread, decreased salt intake occurs without individuals having to consciously engage with any information or actively change their behaviour [9]. So-called “nudge” interventions are one class of lower agency interventions. In the context of changing health-related behaviours, “nudges” have been defined as changes to the placement or properties of objects at the microenvironmental level—for example, changing the placement of healthier options in a buffet [10]. Low-agency population interventions can operate at all levels and not just at the microenvironmental level.

The amount of agency individuals must use to benefit from an intervention is a fundamental determinant of how, and for whom, it will work.

## Intervention “Agency” Influences Intervention Effectiveness and Equity

**Fig 1:**
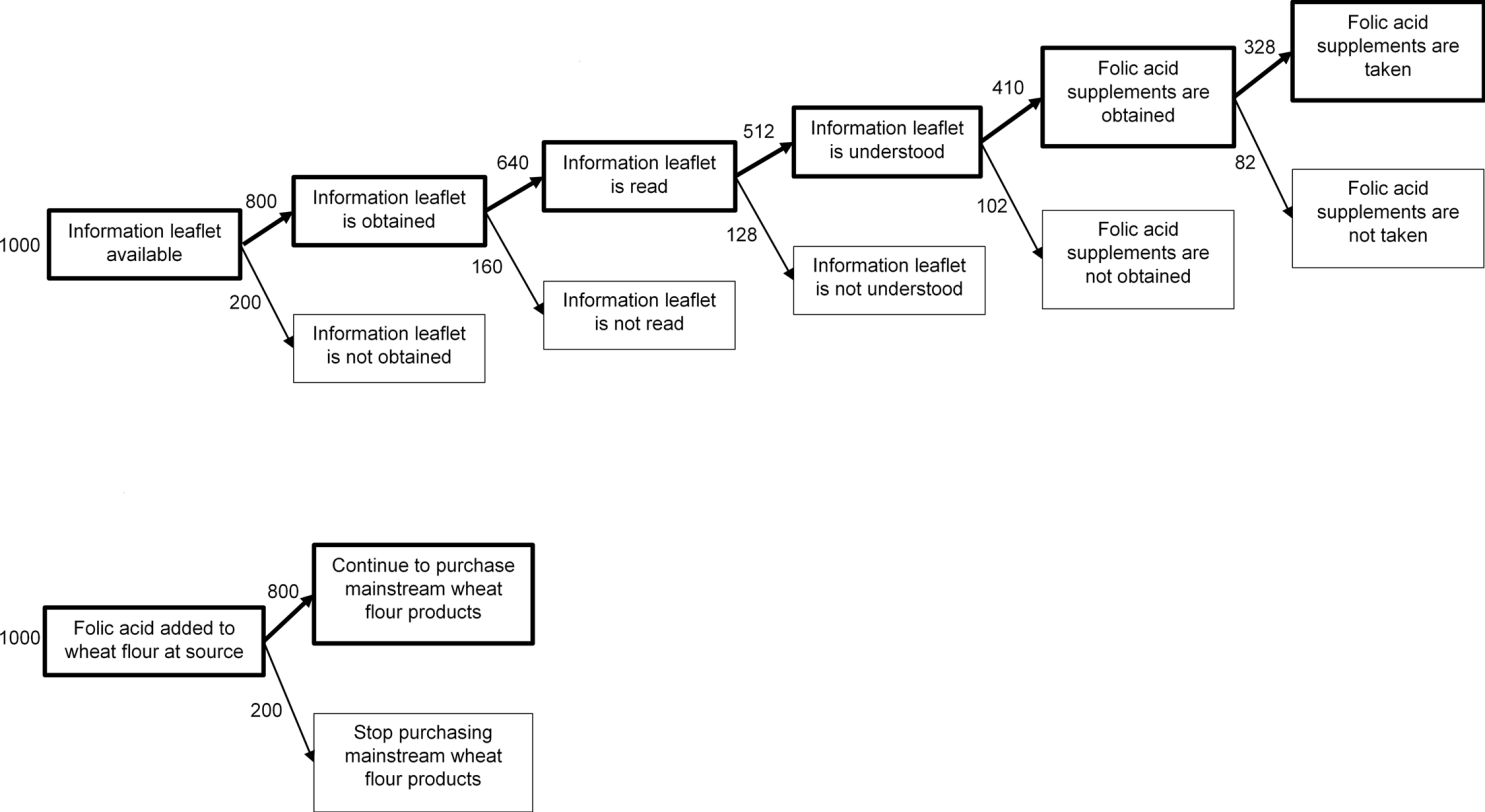
Illustration of the intervention pathway in low- (top) and high-agency (bottom) population interventions. Both examples illustrate population interventions to increase folate in women trying to conceive. The top panel illustrates an information leaflet encouraging women to take folic acid supplements (a high-agency population intervention). The bottom panel illustrates the universal addition of folic acid to mainstream wheat flour (a low-agency intervention). Numbers are illustrative and indicate how many women might be in each pathway if it is hypothetically assumed that there is 20% attrition at each step. The steps shown in both cases are illustrative and not necessarily exhaustive.

There is some evidence that high-agency population interventions are less effective, overall, than those that require less agency [7,11,12]. By reducing the need for individual decisions, there is less room in low-agency interventions for attrition at each of the many steps from intervention delivery to health outcome. For example, for an information leaflet encouraging women trying to conceive to take folic acid supplements (a high-agency population intervention) to have an effect, women must see and read the leaflet, understand the information presented, and then purchase and take supplements. Attrition might be expected at each of these steps. In contrast, if all commercial wheat flour is fortified with folic acid (a low-agency population intervention), one of the few, if only, steps at which attrition can occur is the decision to keep consuming products made with commercial wheat flour. This is illustrated schematically in Fig 1. There is also accumulating evidence that high-agency population interventions are likely to reinforce, or even worsen, socioeconomic inequalities in health [13–16]. For example, information campaigns about the importance of folic acid intake during pregnancy (a high-agency intervention) exacerbate socioeconomic inequalities in folate status more than supplementing food with folic acid (a low-agency intervention) [15].

There are a number of reasons why high-agency population interventions may reinforce socioeconomic inequalities. Exerting agency requires individuals to rally their cognitive, psychological, time, and material resources [7]—all of which tend to be socioeconomically patterned. More socioeconomically advantaged people, with better health literacy (a cognitive resource) [17], may find it easier to make sense of the information provided in public health messages. More affluent parents may have more strategies for resisting their children’s “pester power” for less healthy foods (a psychological resource) [18]. More affluent people are more likely to have the material resources to be able to afford more expensive but healthier foods [19] and the time resources to source and prepare them [20]. Small inequalities at each intervention step illustrated in Fig 1 may multiply to become large inequalities in outcomes [16].

## A New Framework for Public Health Interventions

Our examples above focus on diet, including diet’s role in obesity, but all public health interventions could be placed on a continuum that describes the amount of agency individuals must use to benefit from an intervention. Similarly, while Rose dichotomised preventive interventions as high risk or population, it seems more likely that this too is a continuum. Local planning arrangements restricting proliferation of hot food takeaways near schools target “vulnerable populations” (i.e., children) [8]. This places such interventions somewhere between high-risk interventions targeted at those known to be at high baseline risk and population interventions available to all.

**Fig 2:**
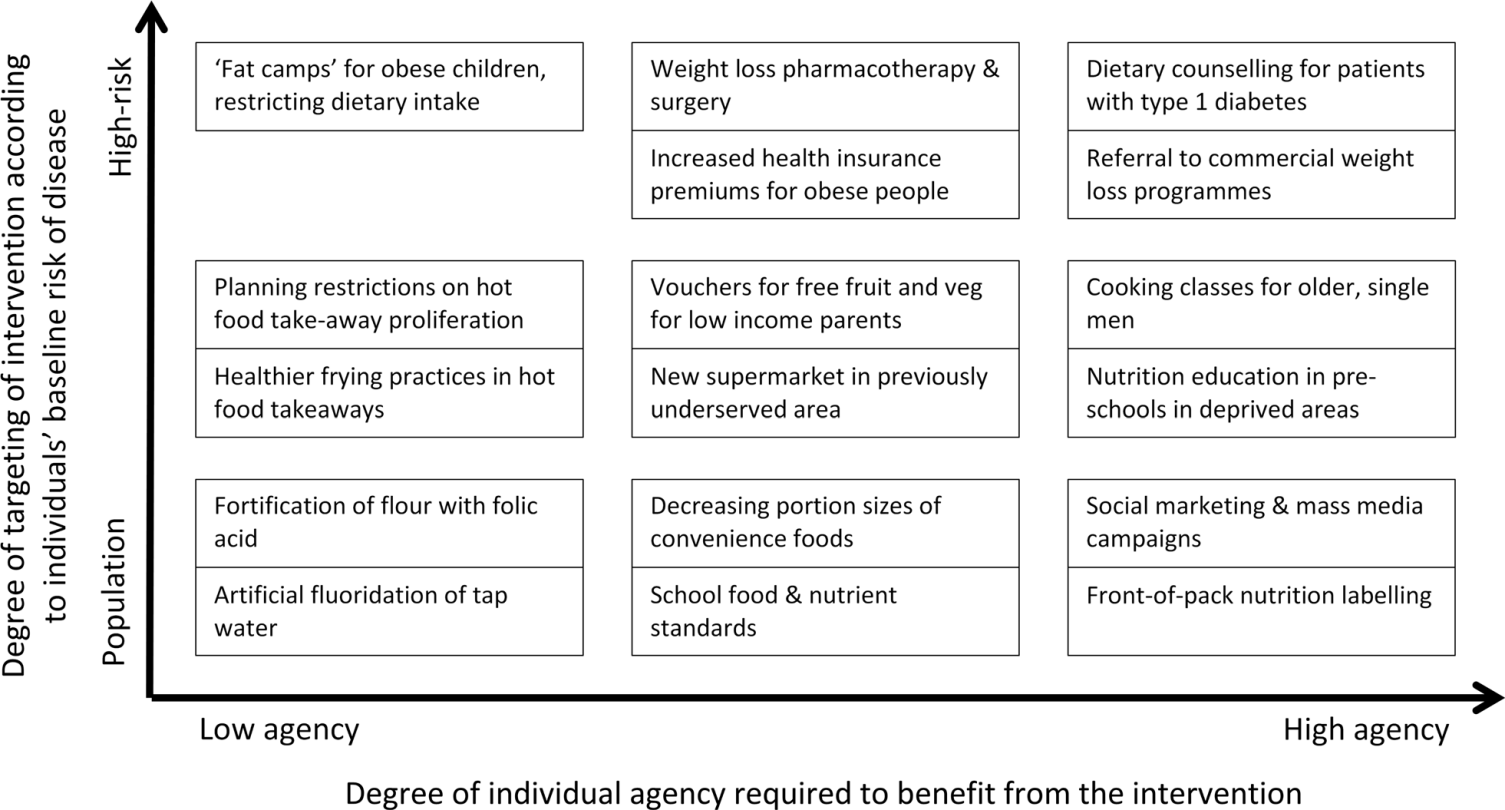
Two continuums describe all public health interventions, with examples related to diet and obesity. Interventions grouped together fall at similar points on the two continuums; we have not attempted to make fine-grained distinctions of where interventions fall on each continuum.

When combined, these two continuums provide a framework for describing the whole range of public health interventions. Fig 2 illustrates this with examples of diet and obesity interventions.

## Low-Agency Population Interventions Should Be the Backbone of Public Health Strategies

Low-agency population interventions are most likely to achieve the twin public health aims of preventing disease and minimising inequalities. These interventions should, therefore, form the backbone of public health strategies.

This is not to say that high-agency population interventions are not valuable. Whilst information and encouragement are often not sufficient to change diet [21], they may still be necessary in some cases. It is also possible that high- and low-agency population interventions act synergistically. For example, a hypothetical intervention involving fortification of flour with folic acid (a low-agency population intervention) that is highly publicised and discussed may raise awareness of the benefits of taking folic acid supplements during pregnancy and so prompt greater engagement with information leaflets about prenatal health (a high-agency population intervention).

## Why Are Low-Agency Population Interventions Underused?

Given the clear value of low-agency population interventions, why do high-agency ones still dominate? One reason may be a perception that low-agency population interventions are less acceptable to various interested parties. In the case of diet and obesity interventions, these parties include politicians, who may enact interventions; the public, who are the recipients of interventions; and food companies, whose commercial interests may be affected by interventions.

Low-agency interventions are sometimes considered to be synonymous with limiting free choice [11]. However, it is unlikely that many people genuinely do make “free choices” about what they eat. Food “choices” are strongly influenced by habits, what food is available and affordable, and cultural norms [22]. Low-income parents often struggle to afford the fruit and vegetables they know to be important for their children’s health [23]. Using subsidies to make healthier food more affordable is a low-agency population intervention that may increase the choices available to these parents.

Public acceptability of low-agency population interventions is dependent not only on how much these interventions are felt to intrude on personal autonomy. Instead, how effective interventions are perceived to be, the importance of the public health “problem” targeted, and personal familiarity with interventions all influence intervention acceptability [24]. England’s experience with banning smoking in public places, in which acceptability increased after the ban was enforced [25], shows that public acceptability of low-agency population interventions is not immutable. The public health community needs to put more effort into learning about, and doing, effective “hearts and minds” work to build public acceptability of low-agency population interventions.

It may seem obvious that low-agency population interventions would be particularly unacceptable to food suppliers, manufacturers, and retailers with vested commercial interests in maintaining current dietary patterns. There is now growing scientific evidence confirming the efforts that those with commercial vested interests can make to avoid low-agency population interventions and the impact these efforts can have on policy [26]. Organisations with vested commercial interests may also have substantial resources at their disposal to circumvent, or undermine, any low-agency public health interventions that are implemented. There is often public cynicism about the actions of food companies [27]. This cynicism could be exploited to give politicians and policy makers the “space” they need to enact low-agency population interventions.

## Conclusion

Much current policy for improving diet and reducing obesity focuses on population interventions that require individuals to use a high level of individual agency to benefit. These are likely to be less effective, and less equitable, than interventions requiring less agency. Although we have focused here on diet and obesity, these arguments ring true across many areas of public health.

Whilst action across the full spectrum of the framework described in Fig 2 is required, more attention should be given to the development and implementation of low-agency population interventions. Many before us have made similar arguments (e.g., [7,21,28]), yet action to implement low-agency population interventions remains limited. Politicians, civil servants, researchers, and others may shy away from low-agency interventions, as they see these as more politically difficult. We all need to have more courage to argue the case that these interventions can be publically acceptable, support people to live healthier lives, and reduce inequalities in health. Low-agency population interventions should be central to public health action on diet and obesity.
