# Correction: Why Are Some Population Interventions for Diet and Obesity More Equitable and Effective Than Others? The Role of Individual Agency

**DOI:** 10.1371/journal.pmed.1002045

**Published:** 2016-05-24

**Authors:** Jean Adams, Oliver Mytton, Martin White, Pablo Monsivais

There is an error in the title of Fig 1. The correct title is: Fig 1. Illustration of the intervention pathway in high- (top) and low-agency (bottom) population interventions.
